# Bi-directional crosstalk between cells and extracellular matrix leads to network morphogenesis in multi-layered tissues

**DOI:** 10.21203/rs.3.rs-2294818/v1

**Published:** 2023-01-31

**Authors:** Youngmin Jo, Donghyun Yim, Chan E Park, Insung Yong, Jongbeom Lee, Wonjin Cho, Kwang Ho Ahn, Chanhee Yang, Jae-Byum Chang, Young-Gyun Park, Taek-Soo Kim, Taeyoon Kim, Pilnam Kim

**Affiliations:** 1 Department of Bio and Braine Engineering, Korea Advanced Institute of Science and Technology, Daejeon 34141, South Korea; 2 Weldon School of Biomedical Engineering, Purdue University, West Lafayette, IN, USA; 3 Institute for Health Science and Technology, Korea Advanced Institute of Science and Technology, Daejeon 34141, South Korea; 4 Department of Materials Science and Engineering, KAIST, Daejeon 305-701, Korea; 5 Department of Bio and Braine Engineering, Korea Advanced Institute of Science and Technology, Daejeon 34141, South Korea; 6 Department of Mechanical Engineering, KAIST, Daejeon 305-701, Korea

## Abstract

Cell-generated mechanical forces drive many cellular and tissue-level movements and rearrangements required for the tissue or organ to develop its shape^1, 2, 3, 4, 5^. The prevalent view of tissue morphogenesis relies on epithelial folding resulting in compressed epithelial monolayers, overlooking the involvement of stroma in morphogenesis^1, 4, 6, 7^. Here, we report a giant web-like network formation of stromal cells in the epithelium-stroma interface, resulting from a multi-scale mechano-reciprocity between migrating cells and their extracellular environment. In multi-layered tissues, surface wrinkles form by a stromal cell-mediated tensional force exerted at the basement membrane. The topographical cue is transmitted to the stromal cell, directing its protrusion and migration along the wrinkles. This inductive movement of the cells conveys traction forces to its surrounding extracellular matrix, remodeling the local architectures of the stroma. In this manner, stromal cells and wrinkles communicate recursively to generate the cellular network. Our observation provides a rational mechanism for network formation in living tissues and a new understanding of the role of cellular-level tensional force in morphogenesis.

Fibroblasts are the primary constituents of connective tissues, accounting for most stromal cells embedded in the extracellular matrix. The fibroblast-derived contractile force is sufficient to generate the deformation of soft tissue^8^. Such a contractile force-mediated deformation has been documented in several systems, primarily in single-layered tissues^3, 9, 10, 11^, but the morphogenesis in a multi-layered biological system remains elusive. In this study, we investigate how stromal cells lead to tissue morphogenesis forming a cellular network at the epithelium-stroma interfaces (ESIs).

First, we visualized the morphology and cellularity of the ESIs in various tissues after whole-tissue clearing, including the intestine, dermis, esophagus, and lung tissue (Extended Data Fig. 1a, and Supplementary Video 1). We observed an aligned cellular network in a three-dimensional en-face view. The stromal cells connected and formed a web-like structure aligned along the direction of grooves (white dotted arrow) (Extended Data Fig. 1a). Unlike previous observations of stromal condensate and follicle patterns at the ESI^4, 7^, the stromal cells were not restricted to the condensate, instead forming a three-dimensional cellular network at the interfaces.

Indeed, the contribution of the stroma is distinguishable during tissue morphogenesis. For example, at gestational week 8^12^, the multi-layered tissue surface emerges with fibrous type I collagen stroma development^13^ (Extended Data Fig. 1b). The critical elements of the dermal-epidermal junction (DEJ) are known to form by 8-10 weeks of gestation^12^, and matured wrinkles in the DEJ are shown in histologies at late gestation compared to that of early stages^14, 15, 16^; similar changes are observed in villi, formed at 9 – 10 weeks of gestation^17^. Thus, the emergence of multilayered tissue is a crucial determinant of the surface morphogenesis of ESI.

To explore the principles of the stromal cell-mediated morphogenesis, we developed a tissue-equivalent model consisting of the epithelium (Epi), basement membrane (BM; reconstructed by Collagen Vitrigel Membrane (CVM), and stroma (Str; fibroblasts embedded collagen matrix) layers (Fig. 1a). The equivalent model formed surface wrinkles, as seen in the tissue (Fig. 1b), and physiologically resembled the layers of the actual tissue in mechano-physical respects (Extended Data Fig. 2). Reconstructing whole layers of tissue consisting of epithelial and stromal cells, we found out that the initially flat surface spontaneously evolved into hierarchical wrinkles with a stromal condensate core (Fig. 1c, white arrowhead, and Supplementary Video 2). The stromal cells were located and aligned with the cellular network along the orientation of the wrinkles. This observation was ubiquitous in other ESIs including ovarian (SKOV3: ovarian cancer cell + NOF: ovarian fibroblasts) and intestinal (SW480: colorectal cancer cells + CCD18Co: colorectal fibroblasts) models (Extended Data Fig. 3a).

Both tri-layered (Epi-BM-Str) and bi-layered (BM-Str) models formed the wrinkled networks (Fig. 1d and Extended Data Fig. 3b). The wrinkle index (*total winkle length / length of projection*) was calculated for each cell type (Fig. 1e). Notably, the wrinkles in BM-Str model is similar in length with *in vivo* tissue surfaces (Fig. 1f). The epithelial and stromal cells formed distinctive wrinkles with different wavelengths and amplitudes, which evolved into hierarchical fractal-like wrinkles when they co-occurred (Extended Data Fig. 3c).

Each layer plays a crucial role in surface morphogenesis. The BM is crucial for wrinkle formation (Extended Data Fig. 3d). As is well-known for the epithelial and BM interaction^7^, the epithelial cells led to invagination, which is greatly enhanced in the presence of BM (Extended Data Fig. 3e). In addition, the epithelial cells contracted basally and apically via phosphorylated myosin light chain II (pMLC2) to induce placode bending resulting in small-scale wrinkle formation. (Extended Data Fig. 3f, g, and Supplementary Video 3). The epithelial cells flattened before basal contraction which enlarged the basal surface and thereby enabled interaction with the BM^6^. The stroma was the key factor in large-scale network formation in the absence of Epi. (Fig. 1g, Supplementary Video 4, 5). Stromal cells formed relatively large grooves that served as the main path in the cellular network (Fig. 1b-f and Extended Data Fig. 3a,b). We therefore sought to elucidate such a new class of morphogenesis driven by stromal cells.

To exclude the contribution of the Epi layer, we conducted bilayered tissue composed of the stromal cell-embedded collagen matrix covered with a thin CVM to act as the BM (BM-Str model). Mouse fibroblasts (NIH 3T3, CellTracker; red) were embedded into the collagen matrix (gray) beneath the CVM (white or green). To observe the onset of wrinkle formation over time, we visualized cells and collagen fibers using fluorescence confocal microscopy and reflection imaging (Fig. 2a, Supplementary Video 6). The cells near the CVM-Str interface extended pseudopods, forming wrinkles in the early period (1.5h) (Fig. 2a). At the initial stage of wrinkle onset, the cells made a proteolytic path while migrating, thereby generating irreversible deformation in the 3D matrix (Fig. 2a inset, Supplementary Video 7). The neighboring cells then moved around the proteolytic path (Extended Data Fig. 4).

At the wrinkle onset, the stromal cells showed polarization of F-actin and phosphorylated myosin II light chain 2 (pMLC2) in the lamellipodia or tips (Fig. 2b). F-actin was concentrated in each tip (white star), whereas MLC2 was highly phosphorylated between the tips (white arrowhead). The cells are attached directly to the CVM interface by focal adhesion molecules, as revealed by paxillin staining. When the myosin inhibitor and blebbistatin (Bleb) were used to block cell contraction before the formation of wrinkles, no wrinkles were produced. By contrast, the contraction inhibition following the development of wrinkles did not affect the pre-formed wrinkles due to the plastic nature of the CVM (Extended Data Fig. 5a). It indicates that the actomyosin-mediated contractility of stromal cells is a key driving factor to form the wrinkle (Fig. 2c).

Using a fibronectin-Forster resonance energy transfer (Fn-FRET) mechanical strain sensor, we examined the strain with high spatiotemporal resolution (Extended Data Fig. 5b,c). The FRET ratio (Ia/Id) decreased (shown by the green pseudocolor) in the regions with high strain. The wrinkle propagated biaxially around the entire interface, leading to changes in the FRET ratio. (Extended Data Fig. 5d, e). This observation was validated with an *in-silico* 3D Agent-Based Model (ABM) comprising contractile cells, fiber matrix, and membrane on the top (Fig 2g, Methods). Membrane deformation *in silico* was quantified and showed noticeable wrinkle formation across two cells (Extended Data Fig. 5f). Particle image velocimetry (PIV) technique was adopted to visualize the surface strain during wrinkling (Extended Data Fig. 5g, h). The displacement map presents the strain field of the surface around the wrinkles. The network pattern defined by the grooves (bulging part in en-face view) exhibited relatively high strain.

Given the fact that cell attachment modifies the local strain and propagates the contractile force across the matrix, cell-to-cell distances influence the wrinkle onset (Fig. 2d, Supplementary Video 8, 9). Cells with a radial distance, *r*, < 70 μm, formed a condensate instantly. However, cells within a range between 70 and 90 μm migrated toward each other, forming condensates. With *r* > 270 μm, the cells did not interact directly but instead formed a long groove that connects two cells (Fig. 2e, f). The principle is validated *in silico* by the ABM (Fig. 2g). As cells were initially located closer, they migrated toward each other while rested stagnant or even migrated away when placed further away (Fig. 2h). Our results suggest that nearby cells are mechanically coupled in a distance-dependent manner and can promote the directional movement of cells along their axis of interaction, forming in topographically confined condensates.

Active cell migration was initiated from the confined condensates within grooves. To migrate, a cell extends protrusions, such as lamellipodia and filopodia, along the wrinkles in response to the topography (Fig. 3a. 8h, Extended Data Fig. 6a). The distant condensates were spread and connected as cell migration continued along the wrinkles (Fig. 3a. 24-32h. white star, Supplementary Video 10). Consequently, most cells were interconnected and aligned in parallel to the wrinkles (Fig. 3b). The aligned cells (lower circularity) within the wrinkle had polarized focal adhesions (Fig. 3c), implying that they generated more traction force than non-stretched cells far from the wrinkles (higher circularity)^18^.

During cell migration, the initial wrinkles were reorganized and evolved into a network. (Fig. 3d, Extended Data Fig. 6b). The two separated wrinkles were joined (black and white star; *joining*). At the junction, a new perpendicular wrinkle formed (black and white arrowhead; *branching*). The phenomena were also validated *in silico* with cell migration (Fig. 3e). In *joining*, the front cells in two neighboring wrinkles (Fig. 3f. i and ii) continued to connect. Combining the two stresses resulted in a wrinkling rearrangement (black dotted arrow) with an acute angle (Fig. 3f, Supplementary Video 11). In *branching*, the migrated cells tugged the membrane in parallel to the wrinkle, forming wrinkles perpendicular to the initial wrinkle (Fig. 3g, Supplementary Video 12). These observations indicate that stromal cells form an interstitial cellular network through repetitive interactions between cell migration and wrinkling.

It is noteworthy that the observed network morphogenesis resembles a hierarchical network of folds in elastic membranes subjected to biaxial compressive stress^19^ in terms of visual appearance and dynamics. In the elastic membrane system, each fold reorganizes the local stress field over time, resulting in a repetitive wrinkle-to-fold transition. Likewise, in our biological model, individual cells generate a force that modifies the stress distribution in spatial and temporal domains, forming a cellular network. It suggests that spatiotemporal force localization and stress relaxation through wrinkling may play a universal role in the formation of the network in layered biological and material systems.

Next, we examined how the cells respond to the dynamic remodeling of the interstitial space. We found that the collagen fibers, filopodia (F-actin) of cells, and wrinkles of CVM were aligned in the same orientation (Fig. 4a). This observation accorded with the results of *in-vivo* analyses. In the dermis, for example, collagen fibers were aligned through the wrinkles under the epithelium interface (Extended Data Fig. 7a). To understand the causality among the aligned components (wrinkle, pseudopod, collagen fiber), we visualized the dynamics of matrix remodeling during wrinkle propagation.

Collagen fiber densification by physical constraint was considered first. The collagen fibers near the cell body were densified in the shrinking groove (Fig. 4b, Supplementary Video 13), whereas those far from the cell body were not (Extended Data Fig. 7b, c). The cell-mediated contractile force is a key driver of the reorganization of collagen fibers (Fig. 4c). The remodeling of collagen fibers near the wrinkles had a reciprocal impact on cell migration, resulting in biased focal adhesion formation depending on the orientation of the fiber segments (Fig. 4d, Supplementary Video 14, 15). Our observations were consistent with previous studies showing that collagen fiber alignment is associated with cell contractile force and migration^11, 20, 21, 22^

Initiation of collagen fiber alignment and subsequent cell protrusion were also observed in interconnecting condensates both *in vitro* and *in silico* (Extended Data Fig. 7d, e). Those fibers were linearly aligned to cell-cell orientation in between cells, while the rest of the fibers around cells are highly aligned themselves, implying the radiating feature of fibers (Extended Data Fig. 7e). In addition, the elongated filopodia occasionally form a tunneling nanotubule (TNT)^23, 24^, resulting in long-distance connections between cells (Extended Data Fig. 7f). These observations reveal that the stromal cells are physically linked through nanoscaled collagen fibers as they modify the local topography of the interstitial matrix. Therefore, the stromal cells can self-organize into networked communities at the tissue interfaces.

Our work demonstrated a new class of morphogenesis driven by stromal cells that form a wrinkle-coupled cellular network at the ESIs. Contrary to the commonly held belief that compressive epithelium forms a folded interface, the tractional force generated by stromal cells is sufficient to alter the interfacial architecture of tissues and thereby trigger directional cell migration, even in the absence of epithelial cells. This phenomenon explains the extensively interconnected cellular network throughout the body^25^.

Our finding also highlighted the important role of loosely connected stromal cells and the BM in organogenesis. Several studies have been performed over the past decade to understand the morphogenesis at the level of multicellular organs, with a particular emphasis on how cell-mediated tractional force can trigger surface morphologies in tissues^1, 3, 4, 13^. In this respect, our results suggest that the traditional view of fibroblasts as a modulator should be modified to reflect their key role in driving morphogenesis at the ESIs.

## Methods

### CVM Fabrication

CVM was fabricated by several steps. First, a premixture of CVM was made by 0.25 mg/ml collagen I (354249, rat tail), 2.3% (to col I volume) of 1M NaOH, 10% (to total volume), 10x PBS, 50% (to total volume) DMEM without serum, 0.05% of Col-F (6346) solution and distilled H2O for the last. A round glass plate was spin-coated with cellulose acetate (CA, 180955, 10 mg/ml) at 1000 rpm 60 sec to form a 110 nm^26^ thickness sacrificial layer. Then donut-shaped PDMS rings with 8mm and 12mm of inner and outer diameter with 300μm thickness were oxygen plasma treated. After the plasma treatment, PDMS rings were placed on the CA-coated glass. The premixture of CVM was poured 122μl in each PDMS ring and incubated at 37°C for 2 hours. After the incubation, the gelled collagen gel was vitrified in a refrigerator with 50% humidity and 18°C temperature for 1 week. Nylon membrane fragments were inserted before gelation for fixed boundaries.

### Materials Modulus Testing

The dog-bone-shaped specimen was gripped by pinning the thin fabric of the specimen in the medium. The real-time load and strain data were obtained by a high-resolution load cell (LTS-50GA, KTOWA, Japan) and DIC technique with a CCD camera (Manta G-504, Allied Vison), respectively. The tensile force was applied to the specimen by the linear actuator (M-111.1 DG, PI, Germany) with a strain rate of 3.2×10^−3^/s.

### Epithelium-BM-Stroma Multilayered Tissue Fabrication

The fully vitrified CVM on the CA-coated glass was rehydrated in DPBS for 10 min. Then the CVM was sterilized with 70% ethanol (in DI) for 10 min. After the sterilization, the CVM on the glass was transferred to an acetone bath. As the CA sacrificial layer was immediately dissolved, the CVM is harvested easily and transferred to the previous 70% ethanol bath. The harvested CVM was inverted and transferred into DPBS. When the CVM is transferred from ethanol to DPBS, it floated around very quickly as the ethanol evaporates. Soon the floating stopped the CVM was transferred onto a flat parafilm. Then 1/50 diluted Matrigel (354234, in DPBS) was coated on CVM by 30 min incubation. After the Matrigel coating, 10mU/ml TR2 (T002, in serum-free DMEM) was coated on CVM for 10 min in RT (※ No washing: as TR2 is a ‘glue’). The adhesive reconstructed BM was ready. The stroma premix (stromal cell embedded collagen I gel premix) should be prepared in the waiting time for the next step. The stromal cell was mixed into col I gel premix in 2x10^5^/ml (for human cells, 1x10^5^ was enough). The col I gel premix was made by 2mg/ml rat-tail col I, 43x1M NaOH to col I, 10x PBS, and the cell containing full DMEM.

The excessive TR2 was removed by pipetting, then the hollow cylinder-shaped PDMS mold coated with Pluronic F-127 (P2443, 0.5% in 70% EtOH) was fitted into the inner hole of the donut-shaped CVM mold. 85μl of the col I gel premix with the stromal cell was poured into the gel mold (※ should be done as fast as possible to prevent evaporation). The mold was incubated for 2h in an incubator for gelation and adherence. After incubation, the gel mold was mildly pressed by a tweezer and the outer CVM mold was removed by striping out. Pouring DMEM would mildly detach the CVM+gel tissue (in gel mold) from the parafilm surface.

To make the full Epi-BM-Str model, the epithelial cell was seeded in 1x10^5^/well directly on the CVM of BM-Str model and incubated at 37°C for 15 min to adhere. After checking the adherence, the whole tissue was transferred into 35pi filled with full media (for the developmental dermis model, add 10ng/ml TGFβ2 (B266458)). The transferred tissue was inverted and agitated smoothly to remove the mold. The free-floating tissue was incubated for the desired time to make self-assembled wrinkles.

To make BM-Str or Epi-BM model, each seeding was omitted.

### Cell Culture

HaCaT was a kind gift from Prof. Shin, KAIST. 3T3, CCD18Co, SW480, NOF, and SKOV3 were purchased from Korean Cell Line Bank. SW480 was cultured in RPMI1640 with 10% FBS and 5% Penicillin/Streptomycin (P/S). All other cell lines were cultured in DMEM with 10% FBS and 5% P/S. To inhibit cell contraction, blebbistatin (B0560) was treated in 10μM concentration with full media.

### Immunofluorescence Staining

4% paraformaldehyde (PFA) was applied to fix samples for 15 min. The blocking and permeabilization reagent (5 % BSA in DI and 0.3 % Triton X-100) was used. After blocking and permeabilization, primary antibodies were attached by overnight incubation in a 4°C refrigerator for 1 hour incubation in RT. The remaining antibody solution was washed out with DPBS three times. The secondary antibodies, including Phalloidin and DAPI, were incubated and washed in the same way. All concentrations of antibodies were according to manufacturers’ instructions. EdU Cell Proliferation Kit (C10640) was used for proliferation assay, done by manufacturer’s instruction.

For live-cell imaging, MitoTracker (M22426) and CellTracker (C34552, C7025, C34565) from ThermoFisher were used in the manufacturer’s recommended concentrations.

**Table:** 

Name	CAS	Concentration
Phalloidin (F-actin)	P1951	1:1000
DAPI	D9542	1:1000
Vimentin	SC-373717	1:200
E-Cadherin	SC-8426	1:200
Collagen IV	ab6586	1:200
Phosphorylated Myosin Light Chain II	3675S	1:200
Paxillin	612405	1:200
Laminin	L9393	1:200
Anti-Mouse 488	F0257	1:200
Anti-Mouse 546	T5393	1:200
Anti-Mouse 640	A21235	1:200
Anti-Rabbit 488	F0382	1:200
Anti-Rabbit 546	T6778	1:200
Anti-Rabbit 640	A21244	1:200
Goat anti Collagen Type IV	Ab769	1:200
Donkey anti-goat Alexa Fluor 546	A11056	1:200
ATTO 647N NHS-ester	AD 647N-31	1:200
ATTO 565 NHS-ester	AD 565-31	1:200

### *In vivo* Tissue Sampling

Experimental animals and all methods were approved by the Korea Advanced Institute of Science and Technology Institutional Animal Care and Use Committee (KAIST-IACUC). Mice used in this study were maintained in a specific pathogen-free facility of KAIST Laboratory Animal Resource Center. C57BL/6J Mice, ages 6–8 weeks, were used in this study. After anesthetization with isoflurane, mice were transcardially washed with ice-cold 1× phosphate-buffered saline (PBS) supplemented with heparin with a concentration of 10 units/mL, followed by perfusion with ice-cold 4% paraformaldehyde (PFA) in 1× PBS. Dermal tissue was harvested and post-fixed with 4% PFA in 1× PBS at 4°C for 6–12 hours. Dermis, intestine, and ovary tissues were treated with SHIELD processing as described previously^27^. Briefly, the heart of a mouse was exposed to perfuse PBS. Then, the SHIELD perfusion solution (20ml for 4min) is applied. After perfusion, each target organ was extracted and incubated in SHIELD perfusion solution for 2 days at 4°C. The organs were incubated in SHELD-OFF solution for 1 day at 4°C. The organs were then incubated in SHELD-ON solution for 1 day at 37°C (epoxy polymerization). After the SHIELD process, the organs were incubated in an SDS clearing buffer for 3~5 days at 47°C. The SDS was washed out by 1% PBSTN for 1 day at 37°C. Finally, The PBSTN was washed out by PBST for 12h at 37°C. The whole organs were treated with an optical clearing solution for 12~24h for transparency.

For expansion microscopy, tissues were first blocked and permeabilized with a blocking buffer (5% NDS (w/w) 0.2% Triton X-100, 1× PBS) for 3 hours. Then the tissues were incubated with the primary antibody in blocking buffer for 2 weeks at 4 °C, and washed in 0.1% PBST 3 times for 30 min each time. Then they were incubated with secondary antibody in blocking buffer for 1 week at 4 °C, and washed in 0.1% PBST 3 times for 30 min each time. AcX was then treated in 1× PBS with a concentration of 0.1mg/mL 6–12 hours at 4°C. The samples were washed in 0.1% PBST 3 times for 30 min each time with gentle shaking. Then the tissues were subjected to a gelation process. In this process, (2.5% (w/w) acrylamide, 7.5% (w/w) sodium acrylate, 0.15% (w/w) N,N′-methylenebis(acrylamide) (BIS), 2 M NaCl, 0.2% (w/w), 0.2% VA-044 (w/w) in 1× PBS) incubated with three times for 6–12 hours each time at 4°C. Then the samples were incubated in a humid chamber at 45 °C for 6 hours for gelation. The gel blocks were then digested with proteinase K diluted to a concentration of 16 U /mL in digestion buffer (1 mM ethylenediaminetetraacetic acid (EDTA), 50 mM Tris-HCl (pH 8.0), 0.5% Triton X-100, 2 M NaCl) 2 times with gentle shaking at 37 °C for 6–12 hours and washing in 0.1% PBST 3 times for 30 min each time with gentle shaking. The gels were stained with diluted fluorescent NHS-ester in 0.2% PBST 6–12 hours with gentle shaking at 4°C. and washed in 0.2% PBST 3 times for 30 min each time. After staining, the skin tissue was homogenized with collagenase type 1 diluted to a concentration of 200U /mL in HBSS buffer 2imes, for 12 hours each time at 37°C. and washed in 0.2% PBST 3 times for 30 min each time. After being digested with proteinase K in digestion buffer 6–8 times at 37°C for 12 hours each time, the samples were treated 2–4 times at 60 °C for 3 hours each time. Fully homogenized gels were stained with DAPI diluted in 1× PBS at 4°C. Then the samples were incubated with an extra volume of fresh deionized water 3 times at 25°C for 15 min each, then overnight at 4 °C for full expansion.

For esophagus and intestine expansion microscopy, tissue sectioning was done. Fixed organs were embedded in low melting (LM)agarose. The (LM)agarose 4% (w/w) was first dissolved and melted in deionized water with a microwave oven at the temperature of 100 °C and then cooled to 40 °C. The organs were embedded in an agarose block by a chamber of cold agarose. The solidified agarose block was then sliced to a thickness of 500–1000 μm with a vibratome (VT1000S; Leica, Wetzlar, Germany).

### Image Processing

3D reconstruction of the confocal image was done with Nikon confocal imaging program and ImageJ. Z-stack projection is done with summation or max intensity projection depending on the data requirement. The raw image was 16-bit, but the processed images were stored in 8-bit or RGB for compatibility with documentation.

### Calculation of the Alignment Percentage

The alignment percentage of subjects A and B were acquired by the difference of orientation between them: D_A-B_. Then the alignment percentage was: (1 − (*D_A−B_*/180)) × 100 %.

### Calculation of the Polarity

The RawIntDen of paxillin in single cell ROI was acquired: PAX_whole_. Then, the RawIntDen of paxillin at the center of the cell was acquired: PAX_DAPI_ (overlapping with DAPI ROI). The polarity was calculated as (*PAX_whole_* − *PAX_DAPI_*)/*PAX_DAPI_*.

### Calculation of the Wrinkle Index

To calculate the wrinkle index, a wrinkle having a certain thickness in the fluorescence image was fitted with a single line. Each wrinkle image sliced along the y-axis was binarized in MATLAB, and the index with the highest fluorescence intensity value was found for the z-axis of each image through the 'findpeaks' function, and then a single line was obtained by connecting them along the x-axis. Then, the wrinkle index was calculated by dividing the single line length of each wrinkle by the image width.

### Find the Importance of Wrinkle Wavelength

To check what wavelength significance appears in the wrinkle made in each cell culture type, classification based on the random forest algorithm was performed. For this, first, the single lines of the previously fitted wrinkle images were Fourier transformed using the ‘fff’ function of MATLAB. After that, classification was performed using the ‘RandomForestClassifier’ function of the python sklearn library. (n_estimator=100, max_depth=1) As a result of this classification, it was confirmed that the accuracy was over 98%, and the 'TSNE' function of the sklearn library was used for dimensionality reduction for visualization. Then the importance of the wrinkle wavelength was calculated by extracting the 'feature_importances_' value from the attributes of the 'RandomForestClassifier' function and taking the reciprocal of the image width.

### FN-FRET

The overall process was according to the previous research^28^. Simply, human plasma fibronectin (33016015) was denatured by 8M guanidine HCl (G7294) and incubated with a 20-fold molar excess of Alexa Fluor 546 Maleimide (A10258). After the conjugation, FN was purified by gel-filtration chromatography from excessive dyes. Then the FN was conjugated by a 65-fold molar excess of Alexa Fluor 488 SDP (A30052) for 1 hour. Finally, the double-labeled FN was purified with chromatography again. The purified double-labeled FN (FN-DA) was stored at −20°C until used. The FN-DA was always used with 90% pure FN to prevent intermolecular energy transfer in fiber.

The fabricated FN-DA was simply mixed with collagen pre-mixture in 50μg/ml^−1^ (5μg/ml^−1^ of FN-DA and 45μg/ml^−1^ of pure FN) to form FN-FRET CVM. The mixture was incubated for 2 hours at 37°C for gelation, then 1 week at 18°C for self-assembly (vitrification).

To measure the relative FRET analysis, two image sets were acquired in confocal microscopy. Two photomultiplier tubes (PMTs) were used to acquire the donor and acceptor signals. The first set of images was donor to donor (DD). The sample was excited with the 488 laser and PMT received 515–525nm filtered wavelength for the donor channel. The second set of images was donor to acceptor (DA). The sample was excited with the 546 laser and PMT received 567–577nm filtered wavelength for the acceptor channel. Finally, DA was divided pixel by pixel by corresponding DD to acquire the FRET image in ImageJ as described in the Extended Data Figure 3c.

The FN-FRET CVM was confirmed by both strains and denature tests (Extended Data Figure 3c). Briefly, the FN-FRET CVM was made on polyethyleneimine-glutaraldehyde (PEI-GA) treated thin PDMS film (300μm thick). The PEI-GA coating provided stable adhesion of protein to PDMS even in extreme strain. Then the CVM-coated PDMS film was stretched by custom zig in 0, 0.2, and 0.6 strains. DA and DD image sets were acquired for each strain to calculate FRET data. Also, the FN-FRET CVM was denatured by GdnHCl in 0M, 1M, and 4M each with 10 min. After the denaturation, DA and DD image sets were acquired for each GdnHCl concentration to calculate FRET data.

### Matrix displacement analysis

For stromal monoculture tissue (BM-Str), 1μm sized bead (Sigma, L4655) was embedded when manufactured. Several live confocal images were taken. The live image was analyzed by iterative PIV^29^ in the ImageJ plugin, while the image alignment was done by template matching^29^. Both plugins were downloaded from https://sites.google.com/site/qingzongtseng/imagejplugins.

For Epi-BM-Str multilayered tissue, since it was challenging to calculate the cell contractile force that induces collagen gel wrinkling, matrix displacement was measured to indirectly confirm the guidance in which the cellular contractile force was applied centering on the gel wrinkling. First, a series of images acquired at 30-minute intervals during the formation of wrinkling were corrected for drift and aligned via the StackReg plugin in Image J.^30^ Then, the aligned images were analyzed using particle image velocimetry software (PIVlab – Matlab-based open-source code) to investigate the folding direction of the matrix by tracking the stained cells around the wrinkles.^31, 32^ After PIV analysis, outliers and noise in the distribution of vectors were effectively removed through post-processing and smoothing. Consequently, matrix displacement was used as an indicator to figure out the direction of the wrinkle to that of the cellular contractile force.

### Statistics

The unpaired two-tailed Student’s t-test was applied to all data (*: P<0.05, **: P<0.01, ***: P<0.001).

### Agent-based computational model

In the model, fibers and cross-linkers constituting the matrix are simplified by cylindrical segments. The membrane representing CVM is coarse-grained into a triangulated mesh. Cells are simplified into spherical phantom units without physical volume.

#### Langevin equation for Brownian dynamics

The displacements of all elements (the center points of cells, the node points of the triangulated mesh, and the endpoints of fibers and cross-linkers) are determined by the Langevin equation with negligence of inertia:

(1) $$F_{i}-\zeta_{i}\frac{dr_{i}}{dt}+F_{i}^{T}=0$$

where **r**_*i*_, ζ_*i*_, **F**_*i*_, and $F_{i}^{T}$ represent the position vector, drag coefficient, deterministic force, and stochastic force of *i*^th^ point, respectively, and *t* is time. The magnitude of the stochastic force is determined by the fluctuation-dissipation theorem^33^:

(2) $$\langle F_{i}^{T}(t)F_{j}^{T}(t)\rangle =\frac{2k_{B}T\zeta_{i}\delta_{ij}}{\Delta t}\;\delta$$

where **δ,** δ*_ij_*, Δ*t*, and *k*_B_*T* are the second-order tensor, the Kronecker delta, time step, and thermal energy. The drag coefficients of matrix elements are determined by the following approximated form:

(3) $$\zeta_{i}=3\pi \mu r_{c,i}\frac{3+2r_{0,i}∕r_{c,i}}{5}$$

where *r*_0,*i*_ and *r*_c,*i*_ are the length and diameter of segments located between endpoints in equilibrium, respectively, and *μ* is the viscosity of a surrounding medium. The positions of all points evolve every time step using the Euler integration scheme:

(4) $$r_{i}(t+\Delta t)=r_{i}(t)+\frac{dr_{i}}{dt}\;\Delta t=r_{i}(t)+\frac{F_{i}+F_{i}^{T}}{\zeta_{i}}\;\Delta t$$

**F**_*i*_ includes extensional forces to regulate equilibrium lengths, bending forces to maintain equilibrium bending angles, and repulsive forces to account for volume-exclusion effects. Extensional, bending, and repulsive forces are determined by the following potential equations:

(5) $$U_{s}=\frac{1}{2}\;\kappa_{s}\;(r-r_{0}{)}^{2}$$

(6) $$U_{b}=\frac{1}{2}\;\kappa_{b}\;(\theta -\theta_{0}{)}^{2}$$

(7) Ur={12κr(r12−r0,12)2ifr12<r0,120ifr12≥r0,12}

where *κ*_s_ and *κ*_b_ are extensional and bending stiffness, respectively. *r* and *θ* are instantaneous length and bending angle, respectively, and the subscript 0 indicates their equilibrium values. *κ*_*r*_ represents the strength of repulsive force, *r*_12_ is the shortest distance between two neighboring elements, and *r*_c,12_ is a threshold distance below which the repulsive force starts acting.

#### Fibers and cross-linkers in the matrix

Each matrix fiber consists of cylindrical segments connected in series. The length of each segment and an angle formed by two interconnected segments are regulated by Eqs. 5, 6 with extensional stiffness (*κ*_s,F_ = 0.277 N/m) and bending stiffness (*κ*_b,F_ = 8.12 × 10^−20^ N·m), and their equilibrium values are *r*_0,F_ = 1.0 μm and *θ*_0,F_ = 0 rad. Each cross-linker is comprised of two segments. The length of each segment and a bending angle between the two segments are regulated by Eqs. 5, 6 with the extensional stiffness (*κ*_s,x_ = 2.0 × 10^−3^ N/m) and bending stiffness (*κ*_b,x_ = 1.04 × 10^−19^ N·m), and their equilibrium values are *r*_0,x_ = 230 nm and *θ*_0,x_ = 0 rad. The repulsive force acts only between fiber segments. The shortest distance between two neighboring cylindrical segments is calculated as *r*_12_, and *r*_c,12_ is equal to the diameter of the segments, *r*_c,F_ = 60 nm. The repulsive force calculated via Eq. 7 with *κ*_r,F_ = 1.69×10^−4^ N/m is distributed to two endpoints of each segment as in our previous study^34^.

#### Membrane and its interaction with the matrix

The length of chains between node points in the triangulated mesh is regulated by Eq. 5 with the extensional stiffness *κ*_s,F_ = 0.001 N/m), and its equilibrium value is *r*_0,M_ = 1.0 μm. A dihedral angle formed by adjacent triangles is regulated by Eq. 6 with the bending stiffness (*κ*_b,M_ = 10 × 10^−19^ N·m), and its equilibrium value is *θ*_0,M_ = 0 rad. The node points can be permanently linked to the endpoints of fiber segments if they are located within 380 nm. The repulsive force acts between the matrix and the membrane by considering the shortest distance between fiber segments and triangular elements in the mesh, *r*_12_. *r*_c12_ is equal to the sum of the radius of the fiber segments, *r*_c,F_/2, and the half of membrane thickness, *r*_c,M_/2, The repulsive force calculated via Eq. 7 and *κ*_r,FM_ = 4×10^−4^ N/m is distributed to two endpoints of the fiber segment and three vertices of the triangular element.

#### Cells and their interaction with the matrix

**Figure 2.**
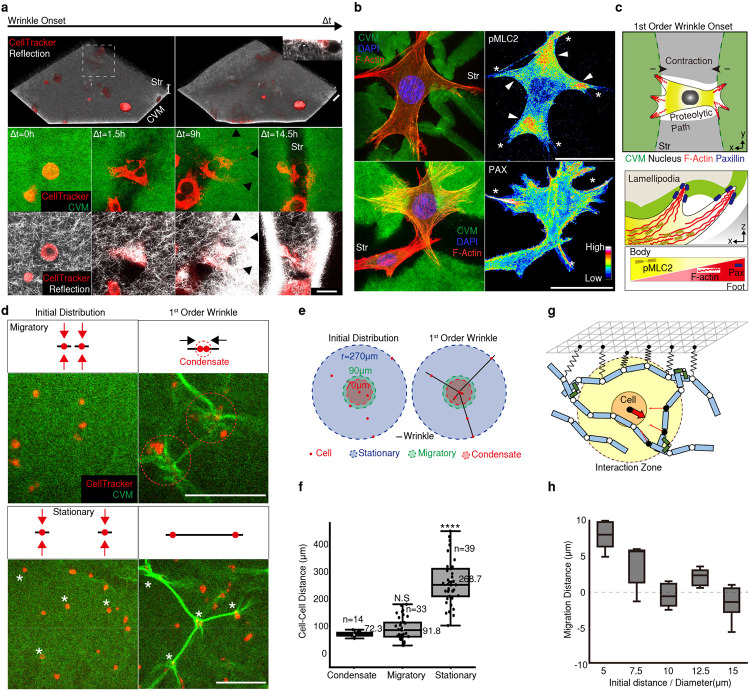
Surface wrinkling is initiated by stromal cells exerting a contractile force on the basement membrane. a) Wrinkle onset overview. A 3D reconstruction showing stromal cells spreading on the CVM interface leaving a proteolytic path (inset) and forming wrinkles. The 2D projection images show that cells anchored to the CVM (black arrowhead) formed wrinkles. Scale bars, 200 μm. b) Focal adhesions (white stars) and motor protein pMLC2 (white arrowheads) showed an umbrella-like contractile wrinkle. Scale bar, 50 μm. c) Scheme of wrinkle onset induced by stromal cell contraction. d) First-order wrinkle formation occurred in migratory or stationary mode. Cell contractile force (red arrow) led to wrinkling (black line). Migratory cells formed condensates (red circle), while stationary cells formed wrinkles connected over large distances (white star). Scale bar, 200 μm. e) Visualized distribution of cells forming different 1’st order wrinkles. f) Quantification of distances in the 1’st order wrinkle. ****, *p* < 0.0001. g) Agent-based computational model mimicking the experimental system. Detailed explanations of the model can be found in Methods. Phantom cells with 2 μm in radius exist in a matrix that consists of fibers (cyan) inter-connected by cross-linkers (green). The matrix is topped by a membrane (triangulated mesh) representing CVM. The membrane is permanently coupled to adjacent fiber points. Cells can be transiently linked to some of the fiber points (black circles) within the interaction zone, then exerting contractile forces (thin arrows) to the fiber points toward cell center points. The sum of reaction forces that cells feel from fiber points (thick arrow) determines the direction of cell movement. h) Dependence of cell migration on initial cell-cell distance normalized by cell diameter. From simulations run with different initial distances between two cells, it was found that cells migrate toward each other only when their initial distance is small enough.

Cells are modeled as spheres, and they can co-exist in the same position with matrix elements (i.e., phantom cells without physical volume) (Fig. 2d, left). Thus, the repulsive force between cells and matrix fibers is not considered. Each cell can mechanically interact with matrix fibers if a distance between the cell center and the endpoint of a fiber segment falls within a range between *R*_C_ and *R*_C_ + *d*_I_ where *R*_*s*_ = 2 μm is a cell radius and *d*_I_ = 4 μm is an interaction distance. All fiber endpoints located within the interaction range can be physically linked to a cell at a constant rate, *k*_+,Fc_ = 0.03 s^−1^. The cell exerts a constant force (100 pN) to fiber endpoints linked to it toward the cell center, and the cell also experiences a reaction force with the same magnitude in an opposite direction. The physical links can be dissociated in a force-dependent manner^35^, following Bell’s equation^36^:

(8) $$k_{-,\text{FC}}=k_{-,\text{FC}}^{0}\;\text{exp}\left(-\frac{|F_{\text{s,F}}|x_{-,\text{FC}}}{k_{B}T}\right)$$

where *k*__,FC_ is a force-dependent dissociation rate, $k_{-,\text{FC}}^{0}$ is a zero-force dissociation rate constant, **F**_s,F_ is a spring force vector on the physically-linked fiber endpoint, and *x*__,FC_ is force sensitivity. Note that it is assumed that physical links behave as a catch bond, so physical links formed on fiber endpoints with high tensile forces last for longer time. The sum of reaction forces acting on each cell is calculated, and the position of its center point is updated using Eq. 1. This leads to cell migration toward high-force or stiffer regions.

#### Domain and its interactions with elements

A rectangular domain (120 × 120 × 60 μm) is employed for all simulations. The boundaries of the domain keep elements within the domain by exerting repulsive forces to elements that attempt to cross the boundaries. It is assumed that the node points of of the membrane on edges and the endpoints of fiber segments close to the boundaries of the domain can be permanently linked to the boundaries. On each boundary, the sum of forces acting on the membrane node points and the fiber endpoints is calculated, then divided by the area of the boundary to calculate stress. By assuming that each boundary represents an elastic material with imposed Young’s modulus, the position of boundaries is updated over time; strain is calculated by dividing stress by Young’s modulus, and then the displacement of the boundary by multiplying the thickness of the elastic material to the calculated strain. The boundary gradually moves toward the displacement level. A rationale for considering the boundaries an elastic material is that a system in our computational model represents a small portion of the in vitro experimental system, so the surrounding elastic material in simulations represents the rest of the real large system.

#### Simulation procedures

For the first 100 s, a simulation system is created via self-assembly of fibers and cross-linkers and the interaction between the matrix and the membrane. The membrane is initially flat and located at *z* = 30 μm, and the matrix is assembled between *z* = 0 μm and the membrane. Cells are allocated in predetermined positions in a symmetric manner relative to the center of the domain; their y and z positions are identical, whereas their x position are 60 μm – *d*c/2 and 60 μm + *d*c/2, where *d*c is an initial distance between two cells. As the value of *d*c, either 20, 30, 40, 50, or 60 μm is used. After creating the system, cells were allowed to interact with surrounding matrix fibers and migrate for 200 sec. The rest of parameters and theirs values are summarized in Extended Data Table 1.

#### Quantification of fiber alignment

To assess the alignment of fiber segments, we first measured alignment for every grid, defined by ⟨cos^2^ *θ* ⟨, where *θ* is the angle between cell-cell orientation and each fiber’s direction and angle bracket averages all angle deviations within a particular grid. As fiber segments are aligned toward cell-cell orientation, alignment gets 1. Furthermore, regardless of cell-cell orientation, order parameter was considered, defined as ⟨2 cos^2^ *ϕ* − 1⟨, where *ϕ* indicates the angle between the mean fiber orientation (of a particular grid) and each fiber’s direction. Order parameter approaches 1 as fibers themselves are aligned in any direction.

#### Data analysis

All graphic representations of results obtained from simulations were created via MATLAB 2021b and VMD^37^.

## Extended Data

**Extended Data Fig. 1.**
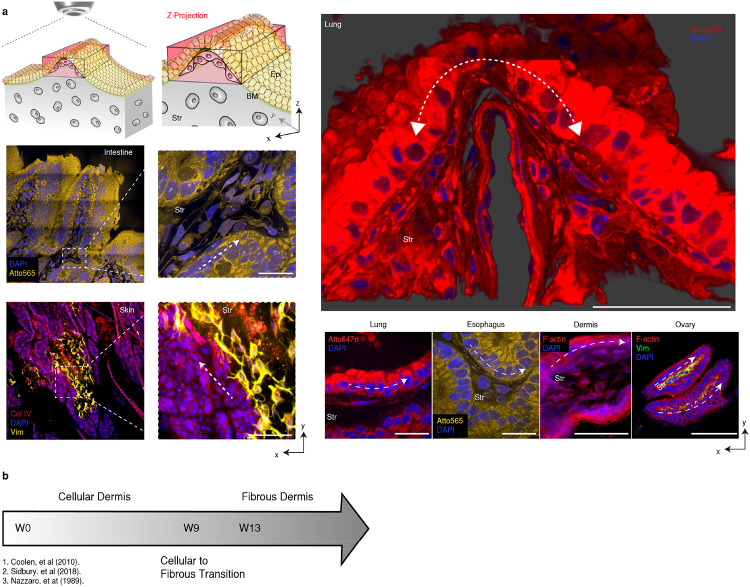
*In vivo* mesenchymal network and surface wrinkles. a) Various hierarchical wrinkles and stromal cells aligned along the wrinkle grooves *in vivo* (white dotted arrow). Scale bars, 100 μm. b) Summary of dermis development by gestation age.^12, 14, 38^.

**Extended Data Fig. 2.**
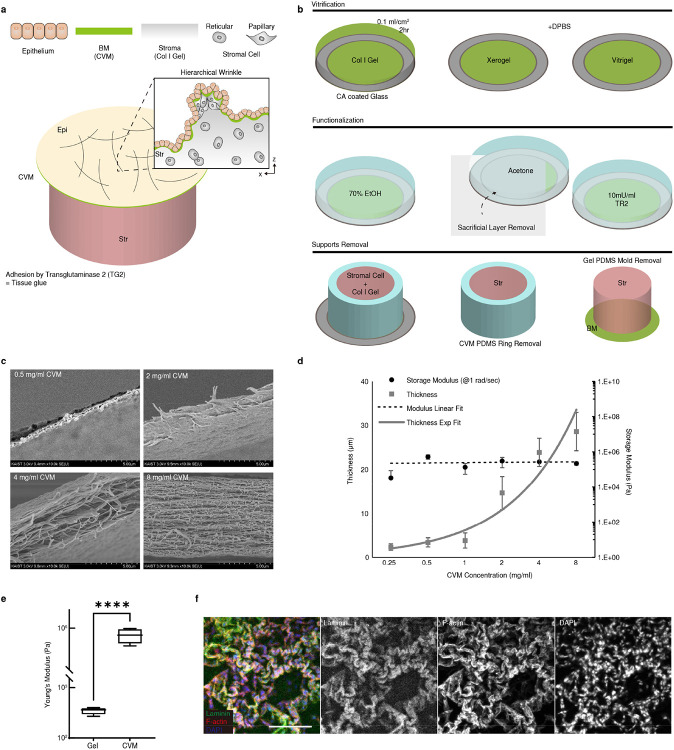
Reconstructed multilayered tissue forming an *in vivo*-like wrinkle structure. a) Schematic view of *in vitro* reconstruction. TG2, a natural glue, was used to adhere CVM to collagen gel. b) CVM fabrication and collagen gel adhesion. c) Scanning electron microscopy image of CVM. d) Thickness and storage modulus according to CVM concentration. e) Young’s moduli of collagen gel and CVM. ****, *p* < 0.0001. f) Laminin expression in the wrinkle. Scale bar, 200 μm.

**Extended Data Fig. 3.**
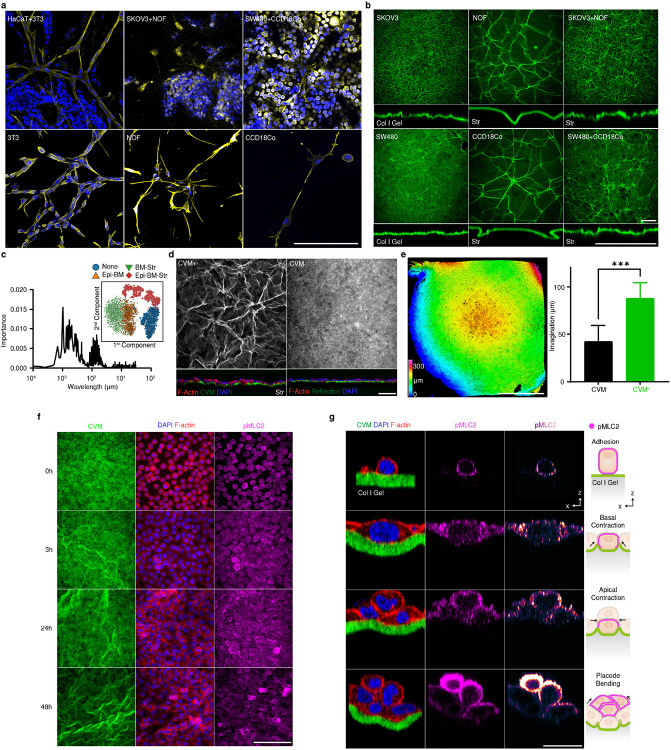
Reconstructed hierarchical wrinkles. a) Mesenchymal networks of various cell types in different tissue types. Scale bar, 200 μm. b) Wrinkles in various cell types in different tissue types. Scale bar, 200 μm. c) Epi-BM-Str model was decomposed with two distinctive wavelengths by machine learning. d) CVM is crucial for surface instability. Scale bar, 200 μm. e) Center invaginated after wrinkle formation in the Epi-BM-Str model. The invagination was deeper when CVM was present. Scale bar, 1 mm. ***, *p* < 0.001. f) Dynamics of wrinkle formation and epithelialization. Scale bar, 100 μm. g) Dynamics of the switch from basal to apical contraction. Scale bar, 20 μm.

**Extended Data Fig. 4.**
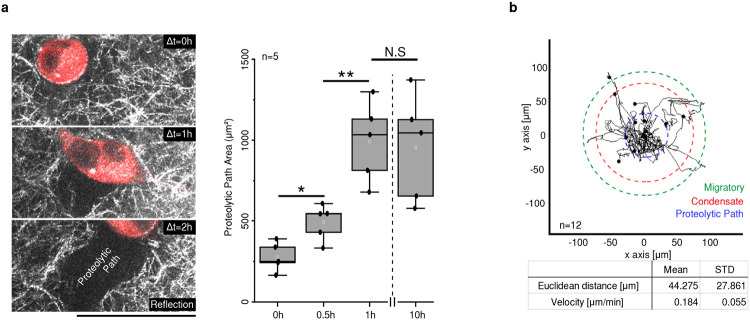
The proteolytic path is the center of migratory wrinkle onset. a) Proteolytic path left behind by stromal cell migration. Scale bar, 50 μm. *, p < 0.05. ***, *p* < 0.001. b) Migration tracking: the proteolytic path, condensate, and migratory area.

**Extended Data Fig. 5.**
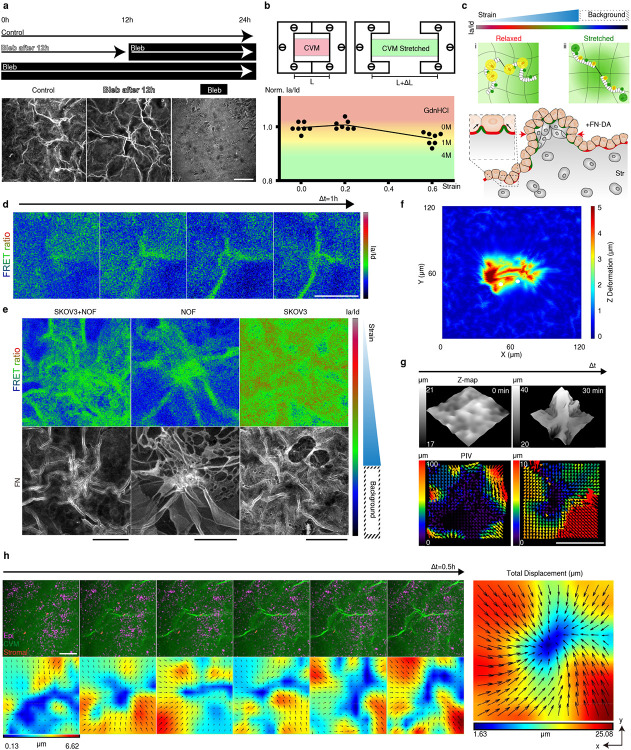
Cell contraction mediated wrinkle onset. a) Contractile force inhibition by Blebbistatin (Bleb). Scale bar, 200 μm. b) The relative Ia/Id value decreased when the Fn-FRET-integrated CVM was under strain on a custom jig or denatured by GdnHCl. c) Scheme of Fn-FRET-integrated CVM. When CVM was under strain, the Ia/Id value decreased. d) Live FRET image of wrinkling showing the effect of strain on wrinkle onset (SKOV3 + NOF). Scale bar, 100 μm. e) The 3D strain pattern of stromal (NOF) and epithelial (SKOV3) cells. Scale bar, 100 μm. f) Membrane deformation in simulations represented by heat map. White circles indicate the locations of two cells beneath membrane. g) PIV analysis during surface morphogenesis in the Str model. Scale bar, 100 μm. h) PIV analysis of Epi-BM-Str multilayered tissue (SKOV3 + NOF). Scale bar, 200 μm.

**Extended Data Fig. 6.**
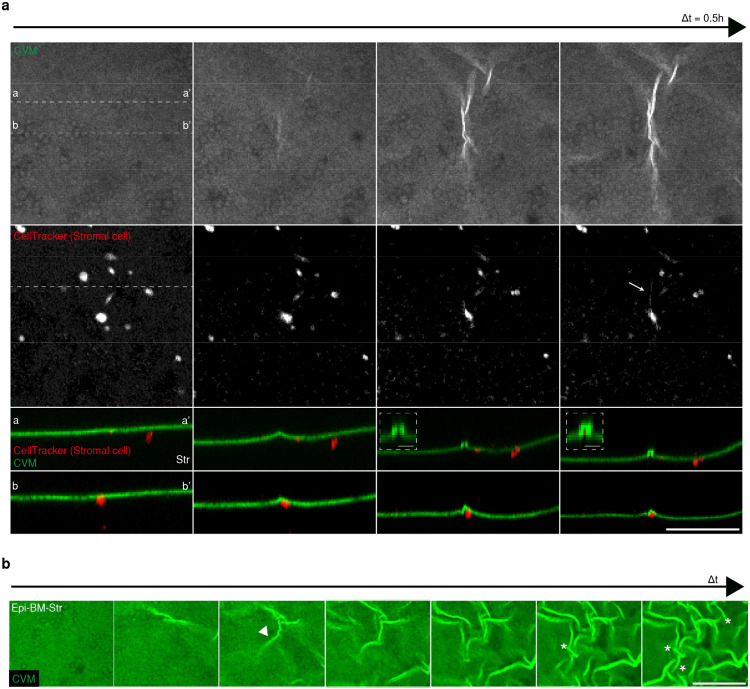
Wrinkle onset and propagation in Epi-BM-Str. a) The Epi-BM-Str coculture model showed wrinkle onset and guided stromal cell migration (SKOV3 + NOF). b) The Epi-BM-Str coculture model also showed wrinkle propagation via *branching* (white arrowhead) and joining (white stars) (SKOV3 + NOF). Scale bars, 200 μm.

**Extended Data Fig. 7.**
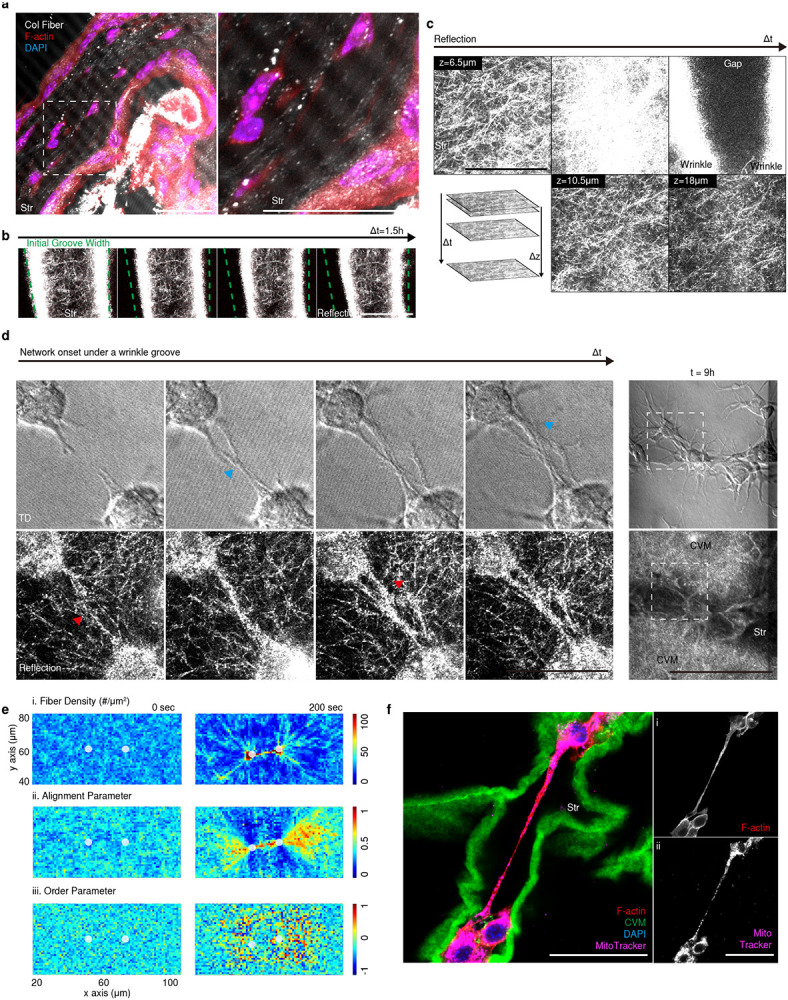
Multiscale paving under wrinkle groove. a) *In vivo* dermal tissue showing the alignment of collagen fibers, stromal cells, and a wrinkle groove. The collagen fiber image was acquired by reflection microscopy. Scale bar, left: 50 μm, right: 10 μm. b) No collagen fiber alignment was seen in the wrinkling area without direct cell contact. The initial groove width (green dotted line) is indicated. Scale bar, 50 μm. c) Collagen fibers under valley (at 6.5 μm), xy plane shifted to the lower z plane (to 18 μm) over time. Scale bar, 50 μm. d) Time-dependent formation of nanoscale fiber alignment and migration. Collagen fibers aligned (red arrow) before cell migration (blue arrow) under the groove. Scale bar, 50 μm. e) Fiber alignment between cells in simulation results shown in top-down view. i: colors indicate the relative density of fiber endpoints counted in each of the 1 μm × 1 μm grids. ii: averaged alignment parameter, given fiber segments’ angle deviations from cell-cell orientation, was used to color each pixel ranging 0 (blue, perpendicular) to 1 (red, aligned). iii: orientational order parameter was shown to visualize the degree to which fiber segments themselves are aligned, where red, green, and blue pixels specifies aligned, disordered, or perpendicular phase. For each row, left columns corresponds to the initial time step and rights for 200 sec. White circles note where two cells are located, f) TNT formation along a wrinkle groove. Scale bar, 100 μm.

## Figures and Tables

**Figure 1.**
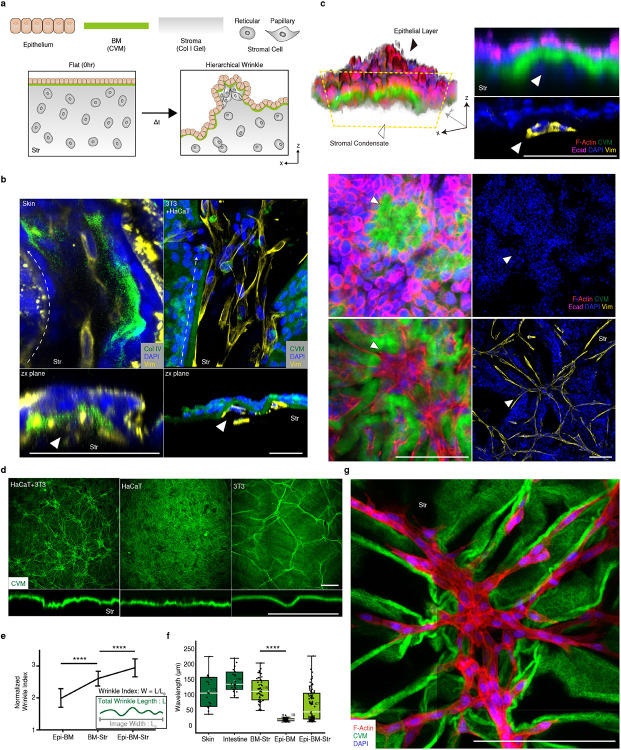
Network of connected stromal cells along the wrinkle structure at the epithelium-stroma interfaces (ESIs) a) Hierarchical wrinkle morphogenesis at the ESIs. b) Stromal cells were aligned (white dotted arrow) under the wrinkle groove, thereby forming a mesenchymal network with stromal condensates (white arrowhead) *in vivo* (left) and *in vitro* (right). Scale bars, 50 μm. Str: Stroma. c) A 3D reconstruction showing the Epi (black arrowhead) and stromal cell condensate (white arrowhead) in the xz plane (top), xy apical plane (middle), and xy basal plane (bottom). Scale bars, 100 μm. d) Epithelial and stromal cells formed distinctive wrinkles. Scale bars, 200 μm. e) Normalized wrinkle indexes differed between Epi-BM, BM-Str, and Epi-BM-Str. ****, *p* < 0.0001. f) BM-Str model replicates the actual tissue surface wrinkle wavelength. g) The larger groove formed by BM-Str led to the formation of the mesenchymal network under the grooves. Scale bars, 200 μm.

**Figure 3.**
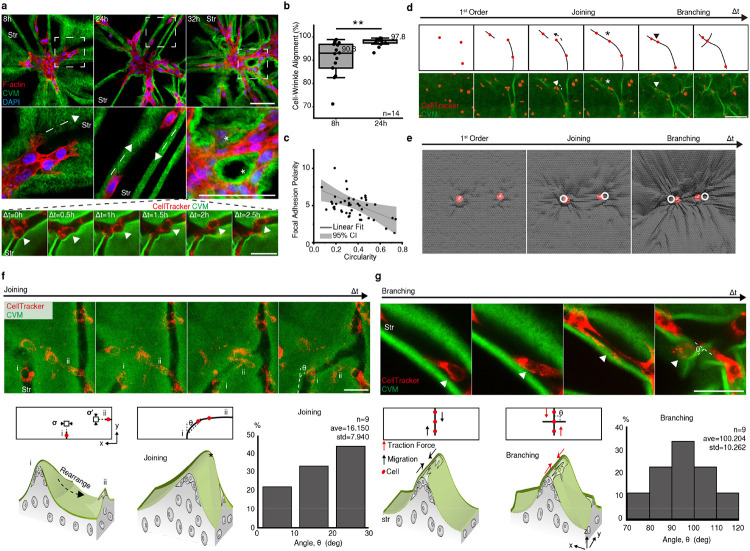
The reciprocal interaction between wrinkle propagation and cell migration permits repetitive *joining* and *branching* of the cellular network. a) Time series of stromal cell migration along the wrinkle groove (white dotted arrow). The migration finally led to the formation of a mesenchymal network by connecting condensates (white stars). Scale bar, upper: 200 μm; lower: 50 μm. b) Coaligned wrinkles and cells. **, *p* < 0.005. c) “Focal adhesion polarity” was inversely correlated with cell circularity. d) Time series of wrinkle propagation. Cells (red dots), wrinkle rearrangement (dotted arrows), wrinkle connection (stars), and migration along the wrinkle (arrowhead) are indicated. Scale bar, 200 μm. e) *Joining* and *branching* behaviors observed on the membrane (gray) in simulations. White dashed lines indicate the initial positions of beads at 0 s. f) *Joining* of two neighboring wrinkles by leading cells (i and ii). θ, degree. Scale bar, 50 μm. g) *Branching* forming a new wrinkle with cell migration (white arrowhead). Scale bar, 50 μm.

**Fig 4.**
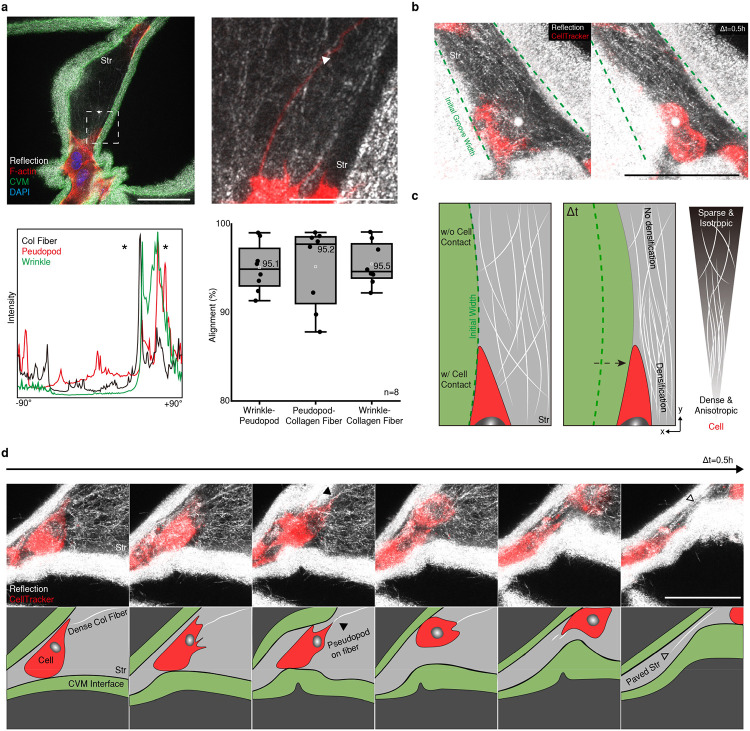
Cell contraction induces multiscale re-organization of a matrix, resulting in guiding and confining the migrating cells into the wrinkle grooves. a) Alignment among a collagen fiber, pseudopod, and wrinkle groove (white arrowhead). The inset line graph shows the orientation alignment (black stars). b) Collagen fiber alignment in the wrinkle area with direct cell contact via compaction. c) Scheme representing collagen fiber densification and alignment gradient around a stromal cell. d) Stromal cells contact collagen bundles (black arrowhead) and migrated through the wrinkle groove, leaving aligned collagen bundles (open black arrowhead). Scale bars, 50 μm.
